# Public Health System Funding for isCGM Reduces Socioeconomic Disparities in Type 2 Diabetes Control: A Cohort Study

**DOI:** 10.1155/jdr/5588397

**Published:** 2025-07-19

**Authors:** Fernando Sebastian-Valles, Jessica Jimenez-Diaz, Carolina Sager-La Ganga, Jon Garai-Hierro, Alicia Justel Enriquez, Alejandra Santamaría Barrena, Jon Portu Gamazo, Luis Eduardo Lander-Lobariñas, María Sara Tapia-Sanchiz, María Ausín Carrera, Julia Martínez-Alfonso, Jose Alfonso Arranz-Martín, Miguel Antonio Sampedro-Núñez, Victor Navas-Moreno, Purificación Martinez-Icaya, Mónica Marazuela, Iñigo Hernando-Alday

**Affiliations:** ^1^Department of Endocrinology and Nutrition, Hospital Universitario de La Princesa, Instituto de Investigación Sanitaria de La Princesa, Universidad Autónoma de Madrid, Madrid, Spain; ^2^Department of Endocrinology and Nutrition, Hospital Universitario Severo Ochoa, Madrid, Spain; ^3^Department of Endocrinology and Nutrition, Hospital Universitario Basurto, Bilbao, Spain; ^4^Department of Family and Community Medicine, Daroca Primary Care Center, Madrid, Spain

## Abstract

**Objective:** The objective of this study is to assess whether the provision of free intermittently scanned continuous glucose monitoring (isCGM) systems can reduce socioeconomic disparities in glycemic control among individuals with Type 2 diabetes mellitus (T2D) treated with multiple daily insulin injections.

**Methods:** This is a cohort study involving 402 T2D patients from three hospitals, all of whom initiated isCGM use as part of routine clinical practice. The isCGM systems were provided free of charge through public healthcare funding, with no out-of-pocket cost to the patients. Glycated hemoglobin (HbA1c) levels were recorded before and after at least 3 months of sensor use. Socioeconomic status (SES) was determined based on the average annual net income per person within the census tract for each patient.

**Results:** Prior to the sensor placement, the mean HbA1c was 8.9% for patients in the lowest SES quartile and 8.2% for those in the highest quartile (*p* = 0.009). Following isCGM implementation, significant HbA1c reductions were observed across all SES groups, with decreases of 1.0% in the lowest quartile and 0.6% in the highest (*p* < 0.001). Postintervention differences in HbA1c between SES quartiles were not statistically significant (*p* = 0.509).

**Conclusion:** Public funding of isCGM systems is associated with a significant improvement in glycemic control and contributes to the reduction of pre-existing socioeconomic disparities in healthcare among T2D patients treated with insulin.

## 1. Introduction

Diabetes mellitus (DM) is a metabolic disorder characterized by chronic hyperglycemia and disturbances in carbohydrate metabolism [1]. Due to its high prevalence and the burden associated with its chronic complications, DM currently represents one of the leading global public health challenges [2, 3]. Estimates of the number of individuals affected by DM exceed 800 million [4]. The vast majority (85%–95%) of diabetes cases in adults are classified as Type 2 diabetes mellitus (T2D) [5]. Notably, the highest prevalence rates of DM in 2022 were recorded in countries within Polynesia and Micronesia, the Caribbean, the Middle East, and North Africa, as well as in Pakistan and Malaysia [4].

Type 1 diabetes (T1D) is an autoimmune condition characterized by absolute insulin deficiency, typically diagnosed in younger individuals, and managed from the outset with intensive insulin therapy [1]. In contrast, T2D is a heterogeneous disease of progressive insulin resistance and relative insulin deficiency, often associated with obesity and aging, and managed initially with lifestyle interventions and oral antidiabetic agents [6]. Insulin therapy in T2D is usually reserved for more advanced stages or cases with poor glycemic control.

Glycemic control, typically assessed using glycated hemoglobin (HbA1c) determination, has been consistently linked to a reduced risk of microvascular and macrovascular complications [7, 8]. In this context, the advent of continuous glucose monitoring (CGM) systems has revolutionized diabetes management. Numerous randomized clinical trials [9, 10] and real-world studies [11, 12] have demonstrated that these devices improve glycemic control in individuals with T1D.

Recently, evidence has also emerged indicating improved glycemic control in large cohorts of patients with T2D [13, 14], including cohorts with patients without insulin therapy [15]. However, access to these technologies may be influenced by economic factors, potentially perpetuating or even exacerbating disparities in glycemic control across different socioeconomic status (SES) levels. Social determinants of health have been extensively studied in T1D, revealing their impact on metabolic control [16, 17], development of complications [18, 19], and even mortality [20–22]. Previous studies from our group showed that free access to CGM systems in individuals with T1D can reduce SES-related disparities [23] in accordance with prior reports in the literature [24].

Nevertheless, there is limited evidence regarding the impact of universal access to these technologies on individuals with T2D. Therefore, the objective of the present study was to evaluate whether the provision of free intermittently scanned continuous glucose monitoring (isCGM) sensors can reduce social inequalities in glycemic control among individuals with T2D.

## 2. Materials and Methods

This is a retrospective pre–post observational study involving a cohort of 402 individuals with T2D on multiple daily insulin injections. Participants were recruited from three Spanish hospitals across different geographic regions: Hospital Universitario de La Princesa (Madrid), Hospital Universitario Severo Ochoa (Leganés), and Hospital Universitario Basurto (Bilbao). All subjects were active users of isCGM systems, specifically the FreeStyle Libre (Abbott).

Inclusion criteria were confirmed diagnosis of T2D and consistent use of an isCGM device, defined as sensor usage exceeding 70% in accordance with current clinical guidelines. Exclusion criteria included diagnoses of T1D, maturity-onset diabetes of the young (MODY), or other specific diabetes forms. HbA1c levels at two different time points were collected, one within 1 month prior to sensor initiation and another one at least 3 months postinitiation, at the time of glucose metrics extraction from the LibreView platform.

The study was approved by the Research Ethics Committee of Hospital Universitario de La Princesa (5917 -04/25) and was conducted in accordance with the principles of the Declaration of Helsinki and the strengthening of the reporting of observational studies in epidemiology (STROBE) guidelines [25].

### 2.1. Procedures

The Spanish National Health System provides individuals with T2D requiring multiple daily insulin injections with isCGM systems at no personal cost upon prescription by a healthcare professional (see Supporting Information section). Participation in a structured education session is mandatory to access the device, and all patients received individualized training on isCGM use prior to sensor initiation, in line with international recommendations [26, 27]. The isCGM system utilizes an electrochemical glucose oxidase sensor implanted subcutaneously, replaced every 14 days. Interstitial glucose readings are wirelessly transmitted to a reader and stored in the cloud via the LibreView platform. Participants were provided with written instructions on interpreting and utilizing sensor data, including real-time insulin dose adjustments, hypoglycemia management, and retrospective data review through LibreView for therapeutic decision-making.

### 2.2. Data Collection

Sociodemographic and clinical variables were retrieved from electronic health records, including sex, age, T2D duration, body mass index (BMI), smoking status, baseline and follow-up HbA1c levels, isCGM usage duration, insulin dosage, number of antidiabetic agents, presence of diabetic nephropathy and retinopathy, and history of ischemic heart disease or ischemic stroke. HbA1c level was measured using liquid chromatography (ADAMS A1c HA8180 V, ARKRAY). Microvascular complications were classified according to international criteria [28–30].

### 2.3. SES

SES was assessed using the mean annual net income per capita of the census tract for each patient, as reported by the Spanish National Statistics Institute [31]. This measure correlates closely [23] with the 2011 Spanish deprivation index [32], but offers a more up-to-date data. Thus, it was utilized as a proxy for SES, analogous to the deprivation index (source: Instituto Nacional de Estadística de España. Atlas de Distribución de Renta de los Hogares 2022. Available in: https://www.ine.es/componentes_inebase/ADRH_total_nacional.htm (visited on 2 April 2025)).

### 2.4. Statistical Analysis

Outliers were examined, and the normality of data distributions was assessed using the Kolmogorov–Smirnov test and normal probability plots. Validated extreme values were truncated at the 1st and 99th percentiles. Continuous variables are presented as mean ± standard deviation (SD), and categorical variables as absolute frequencies and percentages.

Differences in HbA1c across SES quartiles were analyzed using ANOVA and multiple linear regression, adjusting for age, sex, diabetes duration, insulin dosage, BMI, and smoking status.

To evaluate changes in HbA1c over time and the potential modifying effect of SES, we fitted a multivariable mixed-effects linear regression model with HbA1c as the dependent variable. The model included the following fixed effects: time (post vs. pre-isCGM initiation), SES quartile (reference: Quartile 1), interaction terms between time and SES quartiles, age, sex, smoking status (current vs. not), BMI); number of noninsulin antidiabetic agents; and total basal insulin dose per kilogram. The inclusion of time × SES quartile interactions aimed to assess whether the magnitude of HbA1c change following isCGM initiation differed across socioeconomic strata. A random intercept at the individual level was specified to account for within-subject correlation due to repeated measures.

All analyses were performed using STATA v17.0 BE-Basic Edition (StataCorp, College Station, Texas, United States), with statistical significance set at *p* < 0.05.

## 3. Results

**Figure 1:**
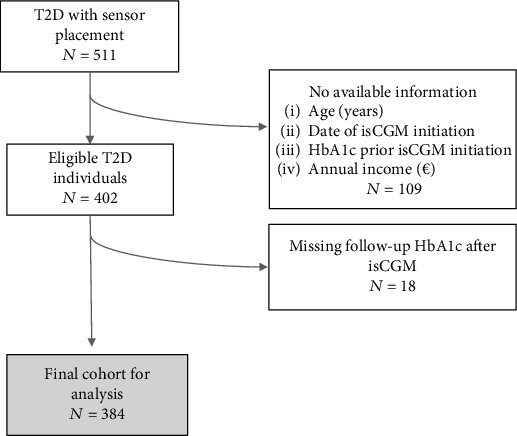
Flowchart. HbA1c, glycated hemoglobin; isCGM, intermittently scanned continuous glucose monitoring; T2D, Type 2 diabetes.

A total of 511 individuals with T2D on insulin therapy who were provided with an isCGM sensor were enrolled. Of these, 402 were selected after exclusion of individuals with missing data for certain variables (Figure 1) with a mean isCGM usage duration of 1.1 ± 0.7 years as of March 2025. The mean age of this cohort was 67.3 ± 10.4 years, and 37.1% of the patients were women (*n* = 149). The mean duration of diabetes was 17.8 ± 4.8 years, and the age at diagnosis was 47.8 ± 10.1 years. The mean BMI was 28.7 ± 4.7 kg/m^2^; 15.3% (*n* = 61) of the patients were current smokers. Diabetic retinopathy was present in 36.3% (*n* = 146) of the patients, chronic kidney disease or microalbuminuria in 54.7% (*n* = 220), a history of ischemic heart disease in 24.6% (*n* = 99), and ischemic stroke in 9.0% (*n* = 36). All complications were assessed prior to isCGM initiation. A total of 384 patients underwent an HbA1c measurement at least 3 months after sensor placement. In this group (final cohort for analysis, Figure 1), HbA1c was reduced after the intervention compared to the basal level (8.5% ± 1.5% [95% CI: 8.4–8.7] vs 7.7% ± 1.1% [95% CI: 7.6–7.8]; *p* < 0.001).

### 3.1. Baseline Differences According to SES

The clinical variables were analyzed across quartiles of net income, which was used as a proxy for SES. Notably, baseline HbA1c levels were significantly higher in the lowest income quartile compared to the highest quartile (8.9% ± 1.8% vs. 8.2% ± 1.3%; *p* = 0.009). Additionally, there was a trend toward higher BMI in individuals with lower income (29.8 ± 4.9 kg/m^2^; *p* = 0.029). No significant differences were observed in other clinical variables across SES quartiles. Detailed results are presented in Table 1.

A multivariable linear regression model was constructed with baseline HbA1c as the dependent variable. The analysis revealed an inverse association between age and HbA1c levels (*β* = –0.023; 95% CI: −0.038 to –0.007; *p* = 0.004). Conversely, female sex (*β* = 0.316; 95% CI: 0.010 to 0.621; *p* = 0.043) and active smoking (*β* = 0.625; 95% CI: 0.205 to 1.046; *p* = 0.004) were positively associated with higher HbA1c values. Neither BMI (*β* = 0.001; *p* = 0.970) nor diabetes duration (*β* = 0.012; *p* = 0.472) showed a significant association. Compared to the lowest income quartile, individuals in Quartile 2 (*β* = –0.509; *p* = 0.016) and Quartile 4 (*β* = –0.505; *p* = 0.020) exhibited significantly lower HbA1c levels; the difference in the Quartile 3 was not statistically significant (*β* = –0.361; *p* = 0.091).

### 3.2. Changes Following isCGM Implementation

**Figure 2:**
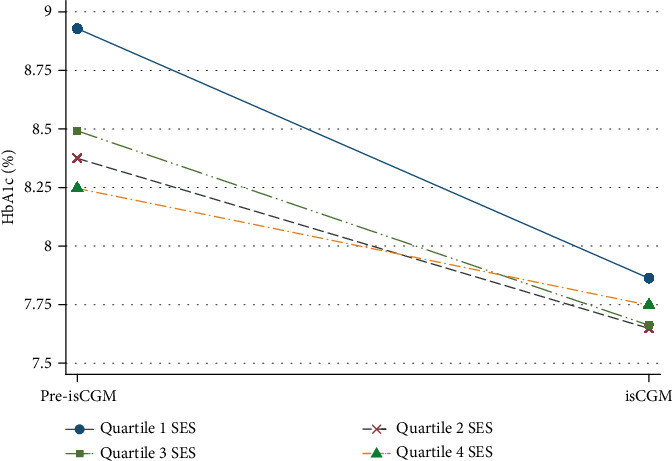
Difference in HbA1c (%) across SES quartiles before and after isCGM initiation. The figure illustrates HbA1c levels before and after the implementation of isCGM in patients with Type 2 diabetes, stratified by SES quartile. HbA1c, glycated hemoglobin; isCGM, intermittently scanned continuous glucose monitoring; SES, socioeconomic status Quartile 1 SES, lowest income; Quartile 4 SES, highest income.

**Figure 3:**
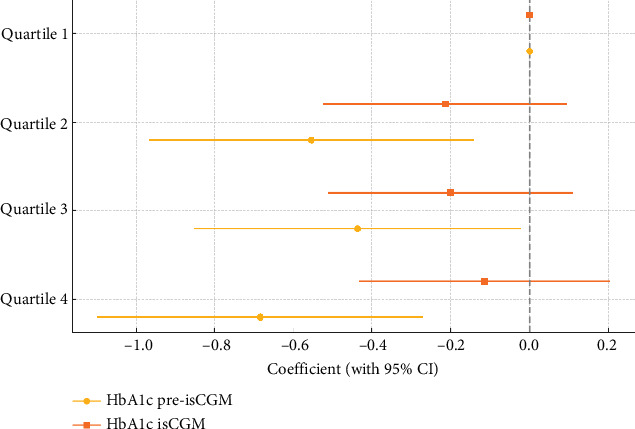
Socioeconomic status-associated HbA1c gradient before and after isCGM implementation. Forest plot displaying the beta coefficients (with 95% confidence intervals) from linear regression models assessing baseline (yellow markers) and postintervention (orange markers) HbA1c levels across SES quartiles, using Quartile 1 (lowest SES) as the reference. HbA1c, glycated hemoglobin; isCGM, intermittently scanned continuous glucose monitoring; SES, socioeconomic status.

HbA1c levels decreased significantly across all SES quartiles after isCGM initiation. In the lowest SES quartile, HbA1c declined from 8.9% ± 1.7% to 7.9% ± 1.0% (Δ = –1.0%; 95% CI: 0.65 to 1.32; *p* < 0.001). In Quartile 2, the reduction was from 8.4% ± 1.6% to 7.7% ± 0.9% (Δ = –0.7%; 95% CI: 0.42 to 1.05; *p* < 0.001); in Quartile 3, from 8.5% ± 1.3% to 7.7% ± 1.0% (Δ = –0.8%; 95% CI: 0.55 to 1.08; *p* < 0.001); and in the highest quartile, from 8.3% ± 1.3% to 7.8% ± 1.2% (Δ = –0.6%; 95% CI: 0.30 to 0.83; *p* = 0.001) (Figure 2). Although all groups experienced a significant improvement in glycemic control, the magnitude of HbA1c reduction was greatest in the lowest SES quartile. In addition, linear regression models showed that lower HbA1c levels were significantly associated with higher SES prior to isCGM initiation, whereas this association was weaker after isCGM implementation (Figure 3). Accordingly, no statistically significant differences in post sensor placement HbA1c levels were observed among SES quartiles (*p* = 0.509), suggesting that isCGM implementation mitigated previously observed disparities in glycemic control associated with SES.

### 3.3. Longitudinal Analysis

A mixed-effects linear regression model with random intercepts at the individual level was applied to evaluate changes in HbA1c over time. Fixed effects included SES quartile, time point (pre/post sensor placement), their interaction, age, sex, smoking status, BMI, number of oral antidiabetic agents, and basal insulin dose per kilogram.

The model revealed a significant overall reduction in HbA1c following isCGM implementation (*β* = –1.04; 95% CI: −1.34 to −0.74; *p* < 0.001). Compared to the lowest income quartile (reference group), individuals in Quartiles 2 and 4 exhibited significantly lower HbA1c levels (*β* = –0.82 and −1.02; *p* = 0.020 and *p* = 0.005, respectively). A significant interaction was observed in the highest SES quartile, indicating a smaller HbA1c reduction over time compared to the reference group (*β* = 0.47; 95% CI: 0.03 to 0.91; *p* = 0.037).

Additionally, active smoking (*β* = 0.42; *p* = 0.007) and higher basal insulin dose (*β* = 0.39; *p* = 0.033) were significantly associated with higher HbA1c levels, whereas older age was associated with lower HbA1c (*β* = –0.01; *p* = 0.034). The mixed-effects model demonstrated significantly better fit than a standard linear model (likelihood ratio test, *p* < 0.001; see Table 2).

## 4. Discussion

The primary aim of this study was to evaluate the impact of publicly funded isCGM implementation on socioeconomic disparities in glycemic control among individuals with T2D treated with multiple daily insulin injections. Our findings indicate that the introduction of this technology was associated with a significant improvement in glycemic control across all socioeconomic strata and a substantial reduction in differences between them.

Numerous studies have demonstrated that lower SES is linked to a higher incidence of T2D [33, 34], poorer glycemic control, suboptimal cardiovascular risk factor management, increased psychological burden [35], accelerated progression of microvascular [36, 37] and macrovascular complications [38], and elevated mortality [39]. In line with this evidence, our study determined a clinically relevant baseline HbA1c difference of 0.7 percentage points between the lowest and highest SES quartiles. A significant trend toward higher BMI in the lower SES groups was also observed. Importantly, the association between SES and baseline HbA1c remained significant even after adjusting for BMI and other covariates in the multivariable analysis. The effect of SES on glycemic control is mediated by several factors. Individuals in socioeconomically deprived areas are less likely to access high-quality healthcare [40]. In our context, limited access to sports and exercise facilities in lower SES neighborhoods has been described, which may partially contribute to differences in obesity and T2D prevalence [41], although our findings suggest that other SES-related factors also play a role. Health literacy tends to be lower in these populations—even in countries with universal healthcare [42]—while unhealthy behaviors such as smoking [43] and high consumption of fast food [44] are more common, further impacting glycemic outcomes.

The benefits of continuous and intermittently scanned glucose monitoring systems have extended beyond their initial indications in T1D [26], with growing evidence supporting their efficacy in T2D [13]. However, their impact on health equity has not been clearly elucidated. Robust evidence suggests that the introduction of new technologies without equitable access strategies can exacerbate existing health disparities [45, 46]. In our study, publicly funded isCGM was associated with a significant narrowing of HbA1c differences across SES groups. These findings are consistent with prior observations in T1D populations [23]. They suggest that universal access to such technologies may help mitigate structural inequities, supporting public health policies aimed at reducing the influence of SES on clinical outcomes. In this context, several countries have begun implementing policies similar to those in Spain to provide glucose monitoring sensors for individuals with T2D. For instance, the 2023 NICE guidelines recommend offering such devices to patients who face difficulties in performing capillary blood glucose testing [47, 48]. Although smoking was not specifically associated with SES in our sample, as reported in other studies [43, 49], it remained consistently linked to a higher baseline HbA1c and less improvement after sensor initiation. Similar findings were also reported in T1D populations [50]. It is worth noting that our study did not assess physical activity. Since this factor is likely associated with both SES and glycemic control, it should be addressed in future research.

There are currently concerns regarding the sustainability of increasing pharmaceutical expenditure in publicly funded systems, particularly those related to the risk of diverting resources from cost-effective interventions toward high-cost technologies with uncertain benefit [51]. However, our findings suggest that isCGM may represent a justifiable investment due to its potential to improve metabolic outcomes and reduce socioeconomic disparities.

This study has several limitations. Its retrospective observational design limits causal inference. Data on physical activity and dietary habits—key factors in glycemic control—were not collected. Although the study was conducted across three tertiary hospitals in different geographic regions, the generalizability of the results to the broader insulin-treated T2D population cannot be guaranteed. Moreover, the SES proxy used was based on census tract income, which may not fully reflect individual-level socioeconomic conditions and could introduce ecological bias. Nevertheless, the use of area-based socioeconomic indicators is well supported in the literature [52–54]. Although in our setting isCGM is universally available to individuals with T2D on multiple daily insulin injections, and the lack of device use is most commonly due to personal preference, we did not include a matched control group of individuals without isCGM. Therefore, we cannot fully exclude the possibility that the observed reduction in SES-related disparities was influenced by unmeasured confounders rather than sensor use itself. Finally, we did not evaluate the impact of SES on other glycemic metrics. This effect should be explored in future studies.

In conclusion, the publicly funded implementation of isCGM in individuals with T2D on insulin therapy was associated with improved glycemic control across all socioeconomic levels and a marked reduction in previously observed disparities. These findings support the role of publicly financed diabetes technologies as a strategic tool to reduce health inequities associated with SES.

## Figures and Tables

**Table 1:** Baseline characteristics of the study sample in the different categories of socioeconomic status.

**Variable**	**Obs, ** **n** = 402	**Quartile 1 income, ** **n** = 101	**Quartile 2 income, ** **n** = 102	**Quartile 3 income, ** **n** = 99	**Quartile 4 income, ** **n** = 100	**p** ** value**
Age	67.3 ± 10.4	67.8 ± 11.7	66.5 ± 10.8	67.5 ± 8.4	67.4 ± 10.5	0.848
Sex, women	149 (37.1%)	42 (41.6)	40 (39.2)	37 (37.4)	30 (30.0)	0.357
Duration of diabetes (years)	17.8 ± 4.8	18.0 ± 3.8	17.3 ± 5.6	18.2 ± 6.0	17.7 ± 3.5	0.608
Net income/person/year (€)	15,379 ± 5260	10,484 ± 1186	12,635 ± 521	15,488 ± 1205	23,015 ± 4223	< 0.001
BMI (kg/m^2^)	28.7 ± 4.7	29.8 ± 4.9	27.9 ± 4.0	28.5 ± 4.4	28.5 ± 5.1	0.029
Smokers	61 (15.3%)	18 (18.2)	19 (18.6)	13 (13.3)	11 (11.0)	0.355
HbA1c (%)	8.5 ± 1.5	8.9 ± 1.8	8.4 ± 1.5	8.5 ± 1.3	8.2 ± 1.3	0.009
Insulin dose (IU/kg)	0.62 ± 0.31	0.58 ± 0.29	0.66 ± 0.30	0.62 ± 0.31	0.60 ± 0.36	0.359
Other antidiabetic agents	2 (1–2)	1 (0–2)	2 (1–2)	2 (1–2)	2 (1–2)	0.315
Retinopathy	146 (36.3%)	46 (45.5)	36 (35.3)	30 (30.3)	34 (34.0)	0.136
Nephropathy	220 (54.7)	54 (53.5)	51 (50.0)	53 (53.5)	62 (62.0)	0.365
Ischemic heart disease	99 (24.6)	21 (20.8)	19 (18.6)	33 (33.3)	26 (26.0)	0.074
Stroke	36 (9.0)	8 (7.9)	10 (9.8)	7 (7.1)	11 (11.0)	0.761

*Note:* Continuous variables are expressed as mean ± standard deviation and categorical variables as *n* (%). Differences between quartiles were analyzed with ANOVA.

Abbreviations: BMI, body mass index; HbA1c, glycated hemoglobin.

**Table 2:** Mixed-effects linear regression model assessing HbA1c variation over time according to SES quartiles.

**Variable**	**Coefficient (** **β** **)**	**95% confidence interval**	**p** ** value**
Time (post vs. pre)	−1.04	–1.34 to –0.74	< 0.001
SES Quartile 2	−0.82	–1.52 to –0.13	0.020
SES Quartile 3	−0.59	–1.29 to 0.12	0.102
SES Quartile 4	−1.02	–1.73 to –0.30	0.005
Interaction: time × Quartile 2	0.30	–0.13 to 0.72	0.169
Interaction: time × Quartile 3	0.20	–0.23 to 0.62	0.361
Interaction: time × Quartile 4	0.47	0.03 to 0.91	0.037
Age	−0.012	–0.023 to –0.001	0.034
Sex (female)	0.19	–0.04 to 0.42	0.104
Smoking	0.42	0.12 to 0.73	0.007
BMI	0.007	–0.019 to 0.032	0.607
Other antidiabetics agents	0.061	–0.049 to 0.170	0.276
Basal insulin (kg)	0.39	0.03 to 0.75	0.033

*Note:* Results of the multivariable mixed-effects linear regression model evaluating changes in HbA1c before and after isCGM initiation, incorporating SES quartiles, time point (pre/post), and their interaction as fixed effects. The model is also adjusted for age, sex, smoking status, BMI, number of oral antidiabetic agents, and baseline insulin dose per kilogram. A random intercept at the patient level was included to account for repeated measures.

Abbreviations: BMI, body mass index; HbA1c; glycated hemoglobin; SES, socioeconomic status.

## Data Availability

The data that support the findings of this study are available from the corresponding author upon reasonable request.

## Supporting Information

Supporting Information Additional supporting information can be found online in the Supporting Information section. Official bulletin issued by the Community of Madrid (June 15, 2023) announcing the public funding of intermittently scanned continuous glucose monitoring (isCGM) systems for individuals with Type 2 diabetes treated with multiple daily insulin injections.
