# Supplementary material for: Public Health System Funding for isCGM Reduces Socioeconomic Disparities in Type 2 Diabetes Control: A Cohort Study
Source: J Diabetes Res. 2025 Jul 19;2025:5588397. doi: 10.1155/jdr/5588397 (PMC12463513; doi:10.1155/jdr/5588397)
Supplement: Supporting Information — Additional supporting information can be found online in the Supporting Information section. Official bulletin issued by the Community of Madrid (June 15, 2023) announcing the public funding of intermittently scanned continuous glucose monitoring (isCGM) systems for individuals with Type 2 diabetes treated with multiple daily insulin injections. [file 5588397.f1.pdf]

Official Bulletin of the Community of Madrid announcing the public funding of glucose monitoring sensors for individuals with type 2 diabetes treated with multiple daily insulin injections (15-JUN-2023)

BOCM BOLETÍN OFICIAL DE LA COMUNIDAD DE MADRID

I. COMUNIDAD DE MADRID

D) Anuncios

Consejería de Sanidad

SERVICIO MADRILEÑO DE SALUD

60 *RESOLUCIÓN de 2 de junio de 2023, de la Viceconsejería de Asistencia Sanitaria y Salud Pública, por la que se hace pública la formalización del contrato del Acuerdo Marco "Sistemas de monitorización continua de glucosa intersticial para pacientes con diabetes mellitus tipo 2 en tratamiento con insulina rápida, para todos los Centros de salud y consultorios locales de la Gerencia asistencial de Atención Primaria del Servicio Madrileño de Salud (lote único)".*

1. Organismo: Servicio Madrileño de Salud.

a) CIF: Q2801221I.
b) Domicilio: Paseo de la Castellana, 280.
c) Localidad y código postal: 28046 Madrid.
d) Código NUTS: ES30.
e) Correo electrónico: licitacionescentralcompras@salud.madrid.org
Dirección de Internet del "perfil del contratante": Portal de la Contratación Pública de la Comunidad de Madrid (http://www.madrid.org/contratospublicos).

2. Tipo de poder adjudicador: Administración Pública-Organismo Regional.
Principal actividad: Salud.
3. Central de compras: Sí, Ley 4/2012, artículo 22, apartado 1.
4. Código CPV: 3319500-3 Sistemas de monitorización de pacientes.
5. Código NUTS del lugar principal de ejecución: ES300 Madrid.
6. Descripción de la licitación:

a) Número de expediente: AM PA. SUM 28/2022.
b) Tipo: Suministro.
c) Naturaleza, alcance y objeto del contrato: El acuerdo marco tiene por objeto la determinación de las condiciones de suministro de sistemas de monitorización continua de glucosa intersticial para pacientes con diabetes mellitus tipo 2 en tratamiento con insulina rápida, que se relacionan en el Anexo 1 del PCAP, cuyas características se especifican en el Pliego de Prescripciones Técnicas, y establecer el procedimiento para su adquisición, mediante contratos basados en este acuerdo, por las unidades y centros dependientes del Servicio Madrileño de Salud relacionados en la disposición adicional tercera apartado 1: Centros y organizaciones adscritos al Servicio Madrileño de Salud: 1.a) Atención Primaria, Centros de Atención Primaria y Consultorios Locales del Decreto 2/2022, de 26 de enero, del Consejo de Gobierno, por el que se establece la estructura directiva del Servicio Madrileño de Salud (BOLETÍN OFICIAL DE LA COMUNIDAD DE MADRID número 22, de 27 de enero de 2022). Las adquisiciones serán efectuadas por la Gerencia Asistencial de Atención Primaria, para el suministro a los Centros de Atención Primaria y Consultorios Locales.
d) División por lotes y número: Sí, lote único.
e) Valor estimado del contrato: 58.277.403,80 euros, IVA no incluido.
f) Presupuesto base de licitación:

— Importe neto: 8.325.825,20 euros.
— IVA (10 %): 832.582,52 euros.
— Importe total: 9.158.407,72 euros.

7. Tipo de procedimiento:

a) Tramitación: Ordinaria.
b) Procedimiento: Abierto.

BOCM-20230615-60

BOCM BOLETÍN OFICIAL DE LA COMUNIDAD DE MADRID

8. Criterios de adjudicación:

— Pluralidad de criterios.

9. Fecha adjudicación del contrato: 31 de marzo de 2023.
Fecha de formalización del contrato: 5 de mayo de 2023.

Empresas adjudicatarias: ABBOTT Laboratories, S. A. CIF: A08099681; Liesno Buno, S. L. CIF: B06982052.

— Importe de adjudicación: Al tratarse de un Acuerdo Marco se adjudica por precios unitarios, no hay importe cierto de adjudicación.
— Ventajas de las ofertas adjudicatarias: Las empresas adjudicatarias cumplen con todos los requisitos exigidos en los Pliegos Administrativos y Técnicos, siendo sus ofertas las más ventajosas por aplicación de los criterios de adjudicación.

10. Procedimiento de recurso:

a) Tipo de recurso: Especial en materia de contratación.
b) Órgano competente: Tribunal Administrativo de la Contratación Pública.
c) Dirección: Plaza Chamberí, 8, 28010 Madrid.
d) Plazo: 15 días, que se computará de acuerdo a lo establecido en el artículo 50 de la Ley 9/2017.

11. Fechas y referencias de publicaciones anteriores:

— "Diario Oficial de la Unión europea": 18 de enero de 2023.
— BOLETÍN OFICIAL DE LA COMUNIDAD DE MADRID: 30 de enero de 2023.
— "Perfil del Contratante": 19 de enero de 2023.
— Fecha envío del anuncio al "Diario Oficial de la Unión Europea": 5 de junio de 2023.

Madrid, a 2 de junio de 2023.—El Viceconsejero de Asistencia Sanitaria y Salud Pública, Fernando Prados Roa.

(03/9.882/23)

BOCM-20230615-60
